# Mapping of Replication Origins in the X Inactivation Center of Vole *Microtus levis* Reveals Extended Replication Initiation Zone

**DOI:** 10.1371/journal.pone.0128497

**Published:** 2015-06-03

**Authors:** Vladimir V. Sherstyuk, Alexander I. Shevchenko, Suren M. Zakian

**Affiliations:** 1 Institute of Cytology and Genetics, Russian Academy of Sciences, Siberian Branch, Novosibirsk, Russia; 2 State Research Institute of Circulation Pathology, Novosibirsk, Russia; 3 Institute of Chemical Biology and Fundamental Medicine, Russian Academy of Sciences, Siberian Branch, Novosibirsk, Russia; 4 Novosibirsk State University, Novosibirsk, Russia; Helmholtz Zentrum München, GERMANY

## Abstract

DNA replication initiates at specific positions termed replication origins. Genome-wide studies of human replication origins have shown that origins are organized into replication initiation zones. However, only few replication initiation zones have been described so far. Moreover, few origins were mapped in other mammalian species besides human and mouse. Here we analyzed pattern of short nascent strands in the X inactivation center (XIC) of vole *Microtus levis* in fibroblasts, trophoblast stem cells, and extraembryonic endoderm stem cells and confirmed origins locations by ChIP approach. We found that replication could be initiated in a significant part of XIC. We also analyzed state of XIC chromatin in these cell types. We compared origin localization in the mouse and vole XIC. Interestingly, origins associated with gene promoters are conserved in these species. The data obtained allow us to suggest that the X inactivation center of *M*. *levis* is one extended replication initiation zone.

## Introduction

DNA replication is required for duplication of the genome and subsequent cell division. First stage of this process is initiation, which occurs at specific sites termed replication origins. About 30,000–50,000 origins are activated in each cell cycle, nonetheless, the total amount of origins is much larger [1]. Several evidences have shown that origins are nonuniformly distributed in the genome and organized into broad zones of replication [2]. Such organization of mammalian origins was reported in earlier studies of the Chinese hamster *DHFR* locus and mouse β-globin and *IgH* loci [3–6]. Although some origins represent well defined isolated replication start sites [7–9] recent genome-wide mapping of origins in human, mouse and drosophila genomes demonstrated that a significant part of the origins is organized into replication zones [10,11]. Origin activation within the zones seems to be stochastic with certain probability or efficiency. However, how origins are regulated within the replication zones and what factors determine origin efficiency are still poorly understood. Origin efficiency is thought to be regulated by chromatin structure. Histone modifications, chromatin remodeling complexes, transcription factors may alter origin efficiency [12,13]. Nevertheless, the impact of various epigenetic features on origin regulation is not well characterized.

In this study, we analyzed replication initiation and chromatin state in the X inactivation center (XIC) of vole *Microtus levis*. XIC is required for inactivation of X chromosome in female mammals and comprises several long non-coding nuclear RNA, among which *Xist*, *Tsix*, and *Enox* are the most conservative [14–20]. During female embryogenesis one of the two X chromosomes is inactivated, whereas the other remains active. A key gene to trigger X inactivation is *Xist*, its transcript coats the entire X chromosome and leads to its heterochromatinization and gene silencing [21–23]. *Tsix* is a negative regulator of *Xist* in rodents and represses *Xist* expression during early embryogenesis [19,24–26]. Transcriptional state of *Xist* and *Tsix* differs between the active and inactive X chromosomes. *Enox* is involved in *Xist* activation and counting of X chromosomes [27]. Although random X inactivation is conservative in eutherian, some differences in this process and its regulation are observed in closely related species such as *Mus musculus* and *M*. *levis* [19,28–30]. Vole XIC is about 60 kb and contains four genes: *Enox*, *Xist*, *Tsix*, and *Slc7a3* [19,31]. *Enox*, *Xist* and *Tsix* demonstrate high sequence similarity with their mouse orthologs. In contrast to mouse, vole XIC lacks a regulatory element, *Xite*, which was replaced with *Slc7a3* gene as a result of chromosome rearrangement. Several origins were previously mapped in a part of the mouse XIC containing *Enox*, *Xist*, and *Tsix* [32,33]. To understand whether these replication initiation sites are conservative in rodents and how chromatin marks in XIC on the active X-chromosome influence origin firing, we analyzed pattern of replication initiation and chromatin state in XIC of *M*. *levis*.

Using qPCR, we analyzed pattern of short nascent strands (SNS) in vole extraembryonic cells—trophoblast stem (TS) and extraembryonic endoderm stem (XEN) cells and in somatic cells—fibroblasts. We found six SNS peaks corresponding to replication origins. Comparative analysis revealed that almost all origins in the XIC are conserved between mouse and vole. We confirmed origin locations within the vole XIC in fibroblasts by ChIP analysis of a subunit of origin recognition complex (ORC). We also analyzed chromatin marks specific to open and closed chromatin states. The data obtained allowed us to suggest that the vole XIC is one replication initiation zone.

## Results

### Mapping of replication origins in the vole XIC in three cell types

**Fig 1:**
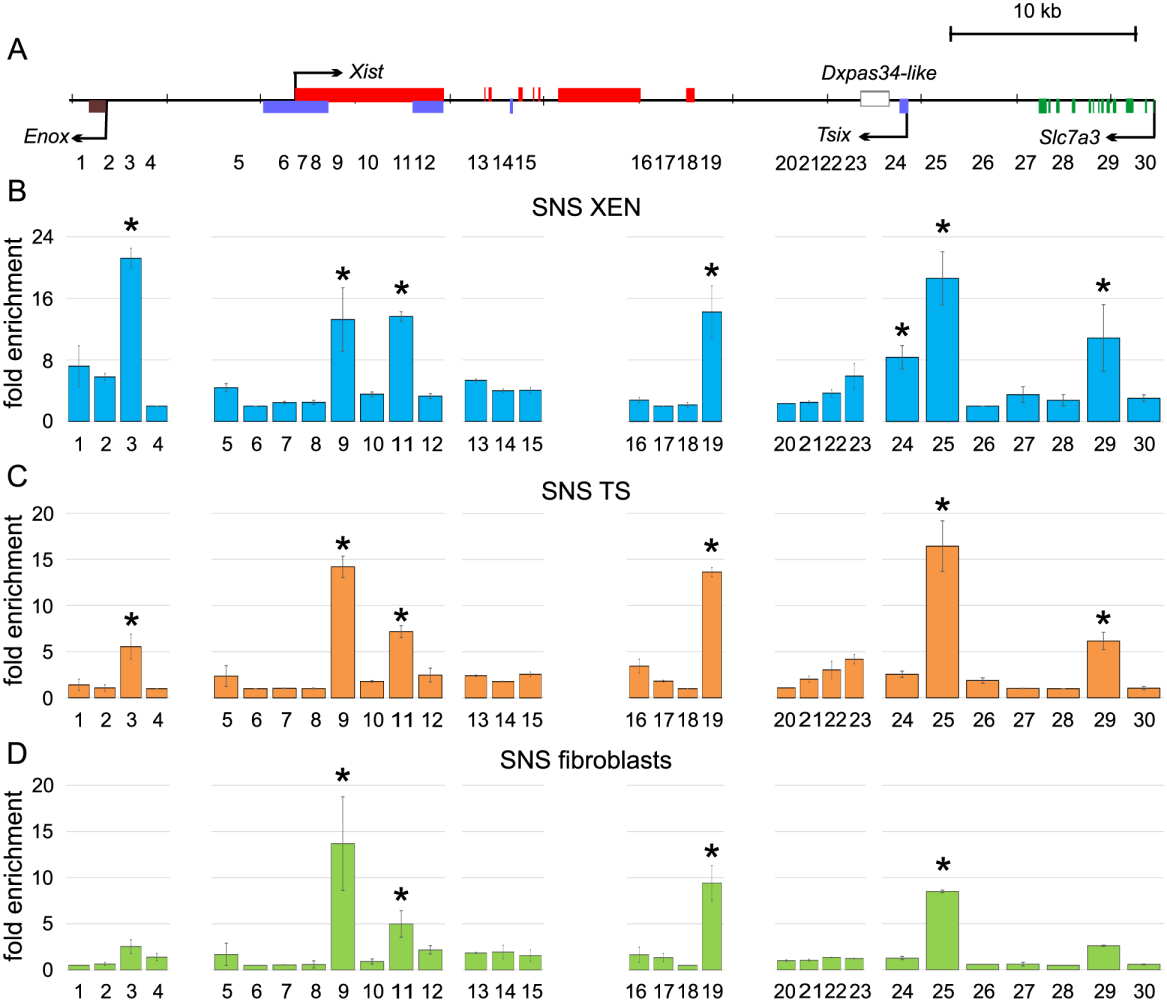
Pattern of SNS enrichment in the XIC locus in XEN, TS cells, and fibroblasts. (A) Schematic representation of XIC locus of *M*. *levis*. Exons are indicated by rectangles. Arrows show direction of transcription. Primer pair locations are shown below. (B, C and D) Pattern of SNS enrichment in XEN cells (B), TS cells (C), and fibroblasts (D). Two independent experiments were performed for each cell line, PCR were carried out in duplicate. ±SD is given. Significant differences * P≥0.95 (one-way ANOVA test).

Nowadays several mapping techniques of replication origins have been developed [34,35]. The most commonly used technique to map replication start sites is analysis of short nascent strands. We used this method to map start sites of DNA synthesis and determine origin activity in the vole XIC. The most commonly used method for SNS purification is centrifugation in neutral sucrose gradient and treatment with λ-exonuclease [34,36]. To compare replication initiation patterns in different cell lines we purified SNS ranging from 750 to 1500 bp from cells representing extraembryonic lineages—XEN and TS cells, and somatic cells—fibroblasts. XEN and TS cells were obtained and characterized previously [29,37,38]. We generated 30 primer pairs and probes located throughout the vole XIC with mean interval of 2 kb except for the repeat containing regions and *Xist* exons 5, 6, and 7 (Fig 1A and S1 Table). Amount of nascent DNA in each region was determined by real-time PCR and normalized to the region that had shown the lowest quantity of SNS. All the cell lines used in this study had normal male karyotype—54,XY. Therefore, all the data were obtained only for one active X chromosome. In XEN and TS cells, we identified six SNS peaks which located near the *Enox* promoter (site 3), in the exon 1 of *Xist* (sites 9 and 11), near the *Xist* 3’ end (site 19), in the *Tsix* promoter (site 25), in the *Slc7a3* gene (site 29) (Fig 1B and 1C). In addition, peak in the exon 1 of *Tsix* (site 24) also shows significant enrichment in XEN cells. In fibroblasts, we identified four SNS peaks that located in the exon 1 of *Xist* (sites 9 and 11), near the *Xist* 3’ end (site 19), in the *Tsix* promoter (site 25) (Fig 1D). Several evidences suggest that origin efficiency is changed during cell differentiation, resulting in different replication initiation patterns in various cell types [10,11,39–42]. We observed vole XIC in fibroblasts contained less active origins than that in TS and XEN cells. In summary, we suggest that vole XIC represents a replication initiation zone that contains five regions demonstrating replication initiation activity.

### ORC binding confirms origin locations in the vole XIC

**Fig 2:**
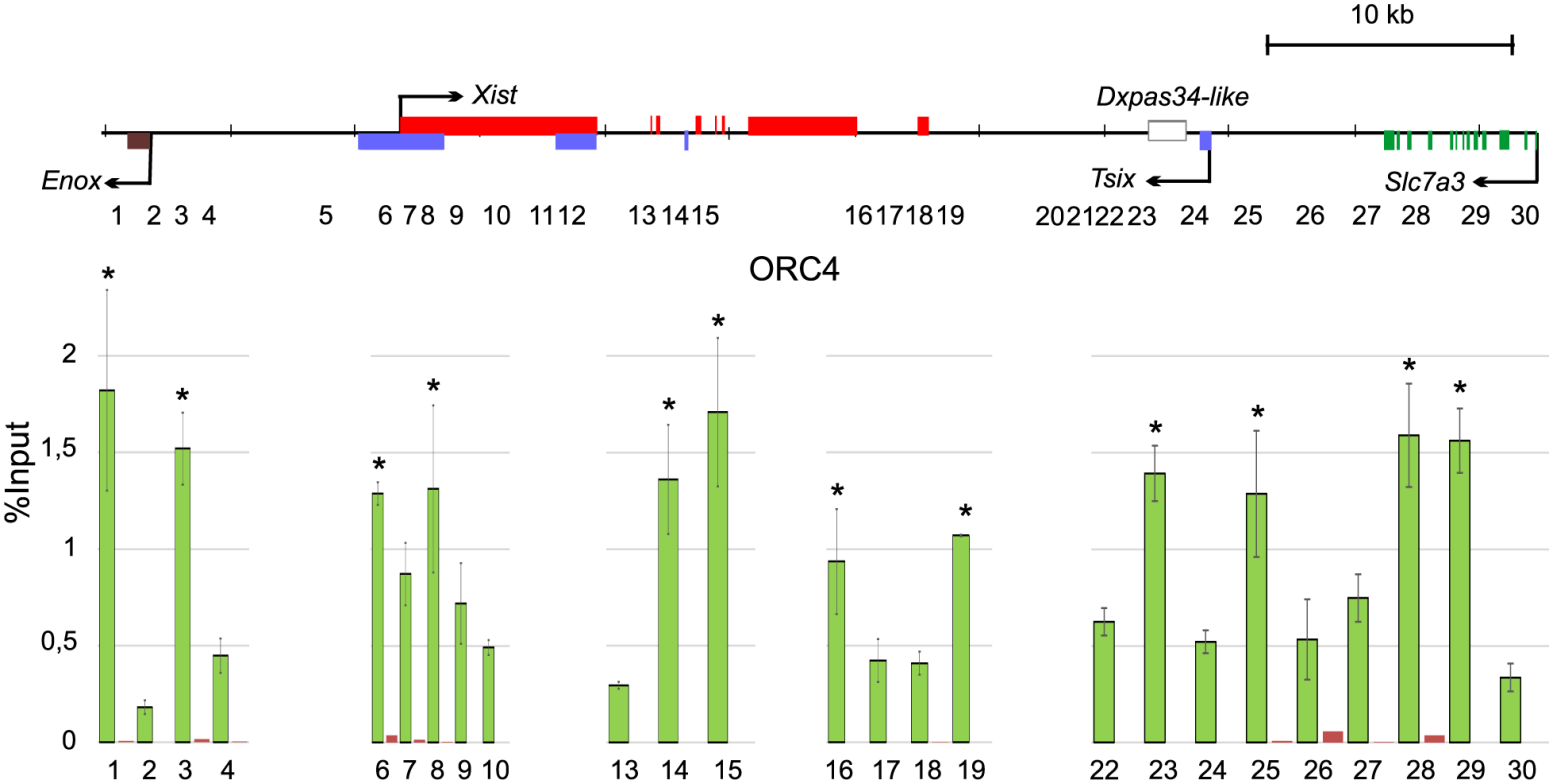
ORC binding regions in the XIC locus. Diagram shows quantitative PCR analysis of ChIP with antibodies to ORC4. Two independent experiments were performed, PCR were made in duplicate. ±SD is given. Significant differences * P≥0.95 (one-way ANOVA test).

To validate origin locations we analyzed ORC binding to the vole XIC in fibroblasts using ChIP approach. ORC is a key component of the pre-replication complex, which is required for origin licensing and activity [43]. We used antibodies to ORC4, a subunit of ORC. We found that ORC4 antibodies were able to recognize the corresponding vole protein (S1 Fig). DNA obtained in ChIP reactions was analyzed by real-time PCR. We identified twelve regions of ORC binding (Fig 2). Several sites are not presented in this histogram because we did not observe any ORC4 enrichment at these sites in a pilot experiment (data not shown). DNA size used for ChIP was less than the distance between different primer pairs so we can assume that neighboring amplicons represent different ORC binding sites. Nine ORC binding regions match the nascent strand peaks or are adjacent to them (sites 1, 3, 6, 8, 16, 19, 25, 28, 29). We also detected ORC binding at the site 24 that was located downstream of the *Tsix* promoter and at the sites 14 and 15 located in the intron 3 and exon 4 of *Xist* correspondingly. Several ORC binding sites did not match SNS peaks and were localized at a distance. Some models suggest that replication initiation may occur at some distance from ORC binding site in the case of expansion of MCM complexes from this site or recruiting MCM to the distal sequences by DNA looping [44]. Several evidences also confirm possibility of replication initiation at a distance from ORC binding site [45,46]. In summary, ORC locations confirm the presence of origins in the vole XIC. In addition, the regions showing replication initiation in the vole XIC contain on average two ORC binding sites.

### Vole XIC comprises high density of G4 motifs

**Fig 3:**
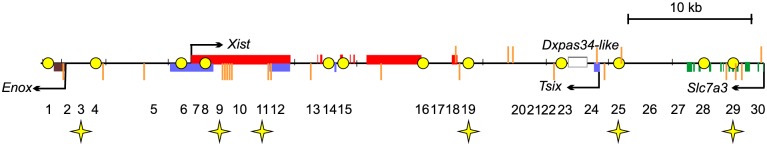
Location of G4 motifs in the XIC locus. Schematic representation of XIC locus of *M*. *levis* and G4 motifs localizations. Exons are indicated by rectangles. Arrows show direction of transcription. Vertical lines show locations of G4 motifs. G4 motifs on the sense strand (according to *Xist* transcription) are shown above, G4 motifs on the antisense strand are shown below. Stars indicate regions containing active origins in all cell lines analyzed and circles do regions of ORC binding.

Recent genome-wide origins mapping studies have demonstrated that a significant part of the human, mouse and drosophila origins is associated with G-rich DNA, which potentially can form G-quadruplexes (G4 motifs) [10,47]. G4 motifs are believed to increase origin efficiency. Another possible explanation for association of replication origins with G4 motifs is that ORC preferentially binds to the regions enriched with G4 motifs. It was shown *in vitro* that human ORC bound preferentially to single-stranded DNA and RNA containing G4 motifs [48]. To investigate association of the nascent strands peaks and ORC binding with G4 motifs in the vole XIC, we analyzed this locus using G4 prediction tool—QGRS mapper [49]. Twenty-five G4 motifs were identified in the vole XIC (Fig 3 and S2 Table). All replication initiation sites were associated with G4 motifs. The highest density of G4 motifs was observed in the *Xist* exon 1, which had origin activity in all cell lines analyzed. This region contained five tandemly located G4 motifs and two of them showed the highest score predicted by QGRS mapper. However, only three of twelve ORC binding regions lied in a close proximity to G4 motifs in the vole XIC (sites 23, 25, 29). Other ORC binding sites were located several kb from the nearest G4 motif. More precise mapping of ORC binding and additional investigations are needed to understand relationships between ORC and G4 motifs or structures in mammals.

### Epigenetic pattern of vole XIC on active X-chromosome

Chromatin structure is believed to regulate choice of active replication origins. One of the mechanisms of this regulation is histone modifications. To determine chromatin structure in the vole XIC, we investigated distribution of histone variant H3.3, H4K20 monomethylation, H3K9 acetylation, and H3K27 trimethylation in XEN, TS cells, and fibroblasts. We also analyzed distribution of histone H3 that was used as a positive control of ChIP reactions. We observed uniform distribution of H3 in XIC except for one site (10) in the *Xist* exon 1, where we detected significant reduction of H3 level in all three cell lines (S2 Fig). We suppose that low H3 level indicates a decreased nucleosome density in this region. Additionally, this region contains DNaseI hypersensitive site in the mouse XIC [50].

**Fig 4:**
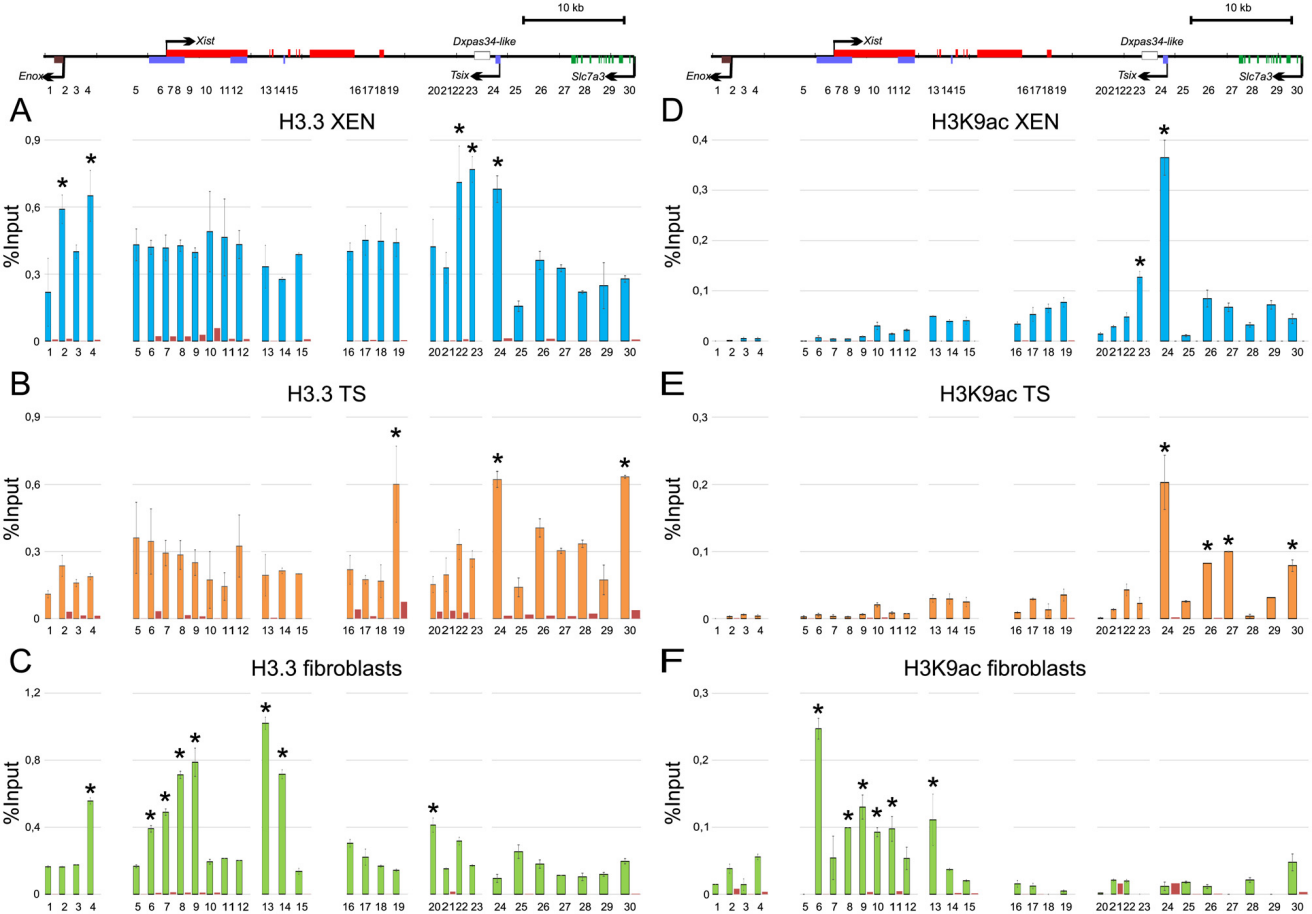
Distribution of H3.3 and H3K9ac in the XIC locus. Diagrams show results of quantitative PCR analysis of ChIP with antibodies to H3.3 in XEN (A), TS cells (B), fibroblasts (C) and H3K9ac in XEN (D), TS cells (E), fibroblasts (F). Red bars represent negative control. At least two independent experiments were performed, PCR were made in duplicate. ±SD is given. Significant differences * P≥0.95 (one-way ANOVA test).

It is known that open chromatin state can promote origin firing. To investigate relationships between localization of origins and open chromatin marks, we analyzed distribution of histone variant H3.3 and acetylated H3K9 in the vole XIC. Acetylation of H3 and H4 histones is believed to enhance origin firing [51–53]. H3.3 is preferentially enriched in the regions of active genes and their promoters [54,55]. In XEN cells we detected statistically significant H3.3 peaks near *Enox* (sites 2 and 4) and downstream of *Tsix* transcription start site (sites 22–24) (Fig 4A). In TS cells, H3.3 peaks were found near the *Xist* 3’ end (site 19), in exon 1 of *Tsix* (site 24) and in the *Slc7a3* 5’ region (site 30) (Fig 4B). In fibroblasts, we observed H3.3 enrichment near *Enox* (site 4), in the promoter and exon 1 of *Xist* (sites 6–9), introns 1 and 3 of *Xist* (sites 13, 14) and in the region located 6 kb downstream of *Tsix* transcription start site (site 20) (Fig 4C). In the exon 1 of *Xist*, H3.3 enrichment demonstrated the highest level 2 kb downstream of the *Xist* transcription start site (site 9) and decreased towards the *Xist* promoter. In XEN and TS cells, the most significant H3K9ac peak is observed in first *Tsix* exon, that is consistent with high expression level of this gene in the extraembryonic cell lines (Fig 4D and 4E and S3 Fig). In contrast to XEN and TS cells, we observed H3K9ac enrichment in the promoter, exon 1, and intron 1 of *Xist* (sites 6, 8–11, 13) in fibroblasts (Fig 4F).

**Fig 5:**
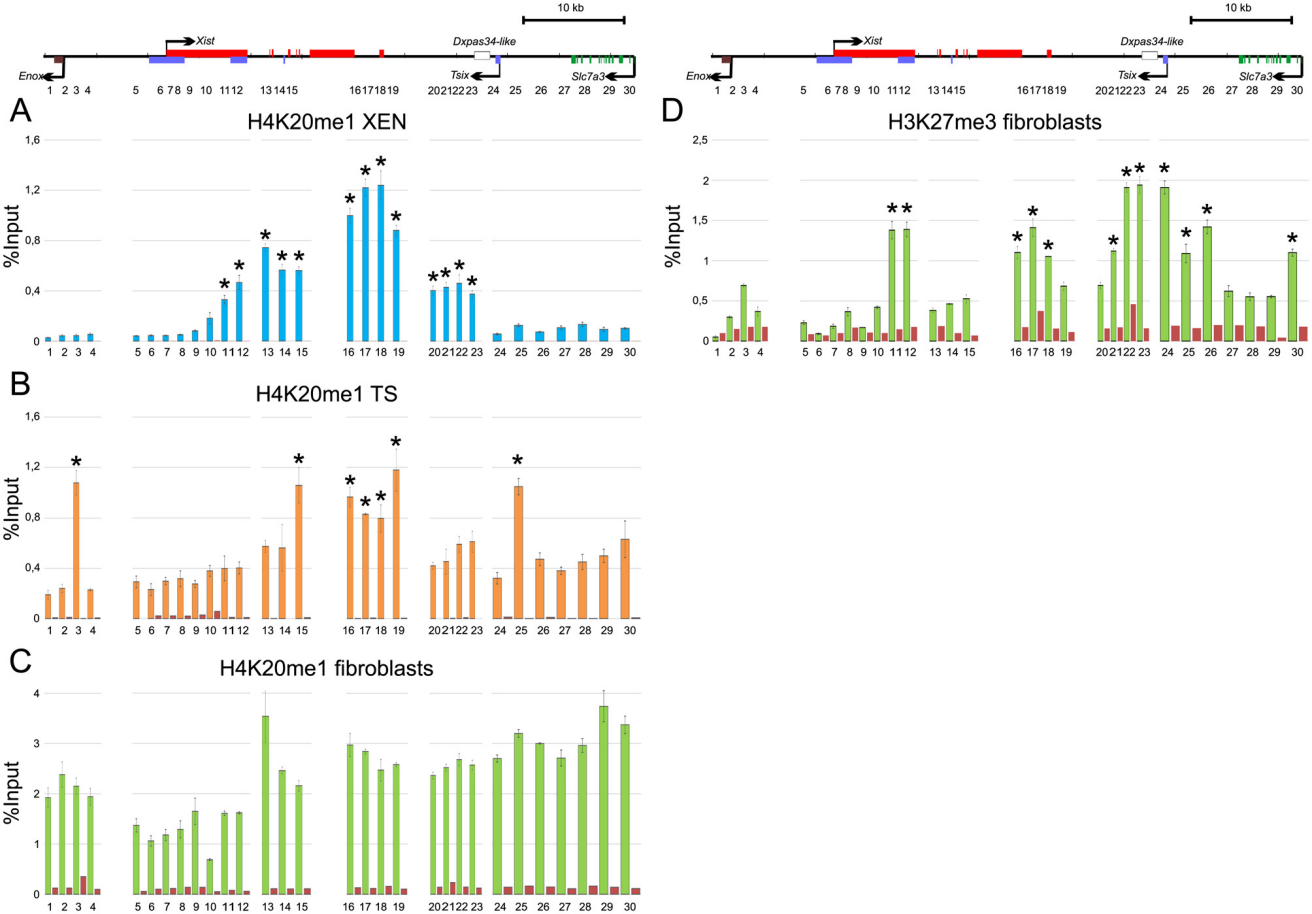
Distribution of H4K20me1 and H3K27me3 in the XIC locus. Diagrams show results of quantitative PCR analysis of ChIP with antibodies to H4K20me1 in XEN (A), TS cells (B), fibroblasts (C) and H3K27me3 in fibroblasts (D). Red bars represent negative control. At least two independent experiments were performed, PCR were made in duplicate. ±SD is given. Significant differences * P≥0.95 (one-way ANOVA test).

We also investigated distribution of chromatin marks that mediate transcriptional repression, such as monomethylation of histone H4 on Lys 20 (H4K20me1) and trimethylation of histone H3 on Lys 27 (H3K27me3). Unlike open chromatin, closed chromatin state may decrease origin efficiency. However, H4K20me1 is suggested to play an important role in origin licensing [56,57]. Moreover, recent findings have shown that H4K20me1 and H3K27me3 are associated with origins activated in mid S phase [58]. We carried out ChIP using antibodies to H4K20me1 and H3K27me3 and analyzed distribution of these marks in the vole XIC. In XEN and TS cells, increased level of H4K20me1 was observed at sites 11–23 and 15–19 correspondingly. In TS cells, H4K20me1 peaks were also observed near *Enox* (site 3) and *Tsix* promoter (site 25) (Fig 5A and 5B). In fibroblasts, H4K20me1 was revealed across the entire XIC locus (Fig 5C). However, the level of H4K20me1 was decreased in the *Xist* promoter, exon 1 and upstream region. H3K27 trimethylation was not detected in XEN and TS cells at this locus. In fibroblasts, H3K27me3 enrichment was found in the 3’ region of the *Xist* exon 1 (sites 11–12), the *Xist* 3’ region (sites 16–18), from region located 5 kb downstream to region located 4 kb upstream of *Tsix* transcription start site (sites 21–26), and in the region located close to the *Slc7a3* transcription start site (site 30) (Fig 5D). Finally, we did not find colocalization of origins with certain chromatin mark in the vole XIC. However, we speculate that different epigenetic marks may act cumulatively on origin regulation.

It is interesting to note that in differentiated murine cells, promoter of the inactive *Xist* allele on the active X chromosome is characterized by trimethylated histone H3 on Lys 9 and hypermethylated CpG dinucleotides [59]. The *Xist* promoter of *M*. *levis*, in contrast to the murine one, seems to have an open chromatin structure in fibroblasts. We suggest that the silent state of vole *Xist* may be maintained by antisense transcription of *Tsix* whose last exon overlaps the *Xist* promoter. Indeed, *Tsix* transcripts were previously identified in vole fibroblasts and other differentiated cell lines [19]. However, this hypothesis needs to be further investigated.

## Discussion

In the last years hundreds of thousands replication origins were identified in the human genome [10,11], few thousands in the mouse one [53,60], and few origins were mapped in other mammalian species [4,61,62]. Mapping and analysis of origins in other mammalian species are needed to compare origins in the homologous regions of the genomes of closely related species. Such an analysis may shed light on regulation of origin efficiency and their choice. In this work, we analyzed pattern of SNS in the XIC of vole *Microtus levis* in three cell types (XEN cells, TS cells, and fibroblasts) and identified that this locus contains six replication initiation sites.

Replication initiation pattern in fibroblasts is slightly different from that in XEN and TS cells. In fibroblasts, XIC contains less active origins. Origin efficiency and activity may change during cell differentiation as has been shown in several works [10,11,39–42].

ORC binding was analyzed in XIC using ChIP approach to confirm origin locations. We revealed at least twelve ORC binding regions. Most ORC binding regions were associated with nascent strands peaks or located proximately. High ORC density in the vole XIC also may imply that XIC represents a replication initiation zone. The most studied replication zone, *DHFR* locus of Chinese hamster, contains large number of pre-RC binding sites and the strongest sites of pre-RC binding do not coincide with preferred sites of replication initiation [63].

Replication origins of higher eukaryotes are not characterized by consensus sequence. However, several genome-wide studies report that a significant part of murine and human origins is associated with G4 motifs [10,47]. G4 motifs can form G-quadruplexes which are involved in the regulation of gene expression in humans [64,65]. We found twenty-five G4 motifs in the vole XIC and most of them was located in the regions that demonstrated initiation of replication or in their close proximity. However, high number of G4 motifs and replication origins in this locus raises the possibility that origins are associated with G4 motifs in the vole XIC accidentally. A genome-wide study of human origins has shown that origin efficiency depends on G4 motif density [10]. Influence of G4 motifs on origin efficiency was also demonstrated for two chicken replication origins [66]. In this case, high number of G4 motifs in this locus may account for high density of replication start sites. We also observed that the highest density of G4 motifs was associated with the origin located to the exon 1 of *Xist*. It should be noted that λ-exonuclease digestion is less efficient on DNA with high GC content [67,68]. The impact of G4 motifs or G4 structures on replication origin efficiency requires additional investigations. Moreover, it has been shown that association of human origins mapped by bubble-seq method with G4 motifs is random [11].

Only part of the total pool of origins is known to be activated in each S phase. It is still poorly understood what factors determine origin choice. Origin firing may be regulated by chromatin structure. We investigated open chromatin marks (histone variant H3.3, H3K9ac) and closed chromatin marks (H4K20me1 and H3K27me3) in the vole XIC in XEN, TS cells, and fibroblasts. It is known that histone acetylation is often associated with active replication origins and may stimulate origin firing [51–53]. H3.3 co-localizes with ORC binding sites in the *Drosophila melanogaster* and *Arabidopsis thaliana* genomes [69–71]. Origin in the *Tsix* promoter was active in all three cell lines whereas the region was acetylated in XEN and TS cells but not in fibroblasts. The opposite situation was observed for the origin in the *Xist* exon 1. In summary, we did not find a strong association of open chromatin marks with origins activity in XIC. We did not reveal ORC association with H3.3 histone variant. Some evidences suggest that only double histone variant H3.3/H2AZ corresponding to labile nucleosomes is colocalized with ORC binding sites [72,73].

H4K20me1 is thought to participate in origin licensing [56,57]. H4K20me1 is enriched in the regions of several origins in XEN and TS cells and present across entire locus in fibroblasts. However, H4K20me1 was not found near *Enox*, in the *Tsix* promoter in XEN cells, the exon 1 of *Xist*, and *Slc7a3* in XEN and TS cells. Genome-wide analysis of human replication origins and H4K20 monomethylation revealed that the histone modification located to the regions with high origin density and colocalized with origins [58]. H4K20me1 was revealed in the significant part of the vole XIC in all cell lines analyzed. It has been shown that H4K20me1 in combination with H3K27me3 have been shown to be associated with origins activated in mid S phase [58]. We found that H4K20me1 and H3K27me3 are colocalized in the regions of two origins in fibroblasts—near the *Xist* 3’ end and in the *Tsix* promoter. Identification of replication timing pattern in vole XIC and its association with chromatin marks analyzed will be subject for further investigations.

**Fig 6:**
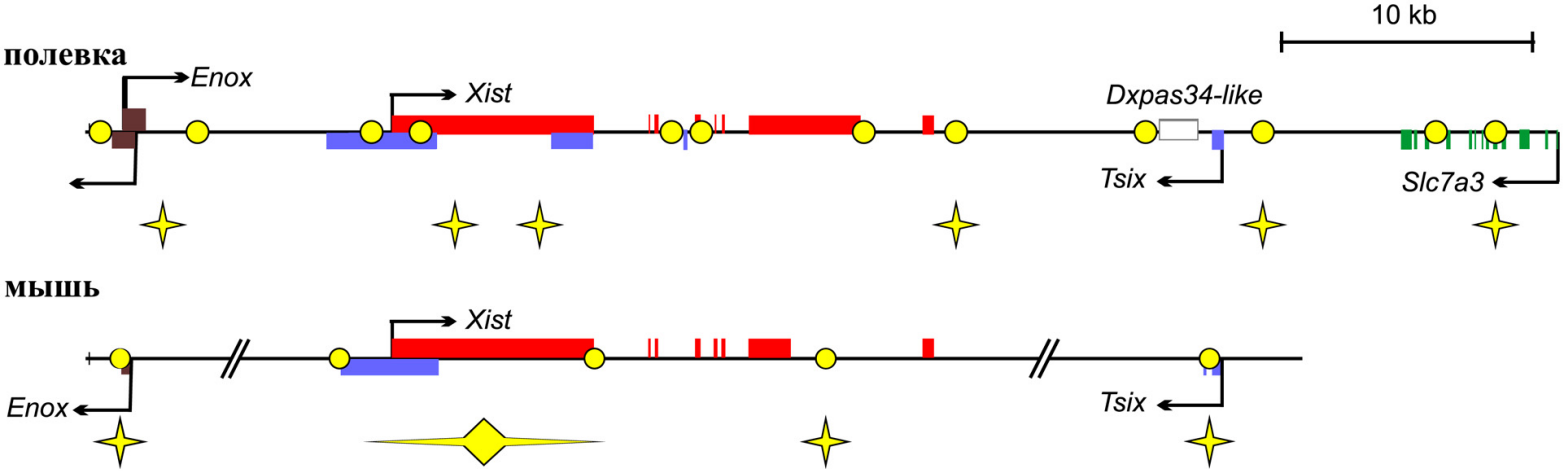
Comparative analysis of replication origin localization in the mouse and vole XICs. Schematic representation of XIC locus with identified replication origins and ORC binding regions in *M*. *musculus* (A) [30] and *M*. *levis* (B). Exons are indicated by rectangles. Arrows show direction of transcription. Stars show regions with active origins in all cell lines analyzed and circles do ORC binding regions.

Several origins were previously mapped in the mouse XIC [32,33]. The origins were located to the *Enox* promoter, the promoter, exon 1 and intron 7 of *Xist*, and the *Tsix* promoter. We assume that origins associated with *Enox*, promoter and exon 1 of *Xist*, and promoter of *Tsix* are conserved in the two rodent species (Fig 6). These origins are associated with functional elements of the XIC despite the differences in nucleotide sequence. Intron 7 of mouse *Xist* also contains an active origin. We also detected origin activity near the 3’ end of vole *Xist*. In mouse XIC, patterns of origin usage in embryonic stem (ES) cells and fibroblasts are similar [33], although some changes were found in replication pattern during ES cell differentiation. Our SNS analysis showed slight difference in replication initiation pattern between the cell lines. We compared replication patterns in cells derived from embryonic and extraembryonic lineages. In mouse, XIC replication initiation pattern was compared in ES cells and their derivatives—fibroblasts. Additionally, ES cells used in this study were maintained in culture medium contained serum. Such an ES cell culture has a primed pluripotency state and its transcription and chromatin profiles differ from the cells of early mammalian embryo [74,75]. Therefore, we believe that changes in replication pattern of XIC locus may occur earlier in development.

To date only few mammalian loci have been described as replication initiation zones. These are Chinese hamster *DHFR* locus, mouse β-globin and *IgH* loci [3,5,6]. Mouse XIC was also proposed to be an extended replication initiation zone [76]. Recent genome-wide studies of SNS and replication bubbles in the human genome have demonstrated that a significant part of origins are organized into replication initiation zones [10,11]. Based on our observations and comparison of replication initiation patterns of the vole and mouse XIC, we suggest that the XIC of *M*. *levis* is a replication initiation zone.

## Materials and Methods

### Ethics Statement

The study was carried out according to “The Guidelines for Manipulations with Experimental Animals.” The study was approved by the Ethical Committee of the Institute of Cytology and Genetics, Novosibirsk, permit number: (order of the Presidium of the Russian Academy of Sciences of April 02, 1980 no. 12000-496). Animals were provided by Animal House Facility of the Institute of Cytology and Genetics SB RAS. Animals were sacrificed by cervical dislocation.

### Cell Cultures

Primary embryonic fibroblasts of *M*. *levis* were derived and cultured as described previously [77,78]. TS and XEN cells were derived and characterized previously and cultured as described [29,37,38].

### Isolation of Short Nascent Strands

Total DNA was isolated with DNAzol from dividing cells according to the manufacturer’s instructions with addition of proteinase K treatment step as described previously [60]. Nascent strands isolation and λ-exonuclease treatment were performed as described previously [60]. DNA was layered onto neutral 5 to 30% sucrose gradient prepared in TEN300 (10 mM Tris-HCl, pH 7.9, 2 mM EDTA, 300 mM NaCl) and centrifuged in a Beckman SW41 Ti rotor at 24000 rpm, 4°C, for 22 h. Fractions were withdrawn from the top of the gradient and a small aliquot of each fraction was run on a 1.2% alkaline agarose gel at 50 V overnight at 4°C. Fractions corresponded to 750–1500 bp were pooled and precipitated with ethanol. Before λ-exonuclease treatment, DNA was phosphorylated with T4 polynucleotide kinase (PNK) (NEB) in 1 × PNK buffer at 37°C for 1 h. After phosphorylation, DNA was precipitated with ethanol. Digestion was performed with 100 U of λ-exonuclease (Fermentas) in 1 × λ-exonuclease buffer at 37°C overnight. After digestion, DNA was extracted once with phenol/chloroform/isoamylalcohol and once with chloroform/isoamylalcohol and then precipitated with ethanol. T4 PNK phosphorylation and λ-exonuclease digestion were performed twice, after final purification SNS were resuspended in water and analyzed by real-time PCR.

### Chromatin Immunoprecipitation (ChIP)

ChIP was performed according to a protocol published previously [79] with several modifications. Crosslinking of 10^7^ cells was carried out by adding formaldehyde (Sigma) to a final concentration of 1% to the culture medium for 5 minutes at room temperature and stopped by addition of glycine (Sigma) to a final concentration of 125 mM. Cells were washed twice with ice-cold PBS and lysed in Lysis Buffer №1 (50 mM HEPES-KOH, pH 7.5, 140 mM NaCl, 1 mM EDTA, 10% glycerol, 0.5% Igepal CA-630, 0.25% Triton X-100, protease inhibitors). Then nuclei were incubated in Lysis Buffer №2 (10 mM Tris-HCl, pH 8.0, 200 mM NaCl, 1 mM EDTA, 0.5 mM EGTA, protease inhibitors) for 10 minutes, and chromatin was sheared in Lysis Buffer №3 (10 mM Tris-HCl, pH 8.0, 100 mM NaCl, 1 mM EDTA, 0.5 mM EGTA, 0.1% Na-deoxycholate, 0.5% N-lauroylsarcosine; protease inhibitors) by sonication into fragments ranging from 100 to 600 bp. 1/10 volume of 10% Triton X-100 was added to sonicated chromatin, cell debris was removed by centrifugation at 14000 rpm for 10 minutes. Supernatant was collected and incubated with antibodies overnight. As negative control of ChIP we performed reaction without antibodies. Dynabeads Protein G (Life Technologies) was used to collect antibody-protein complexes. The complexes were washed five times with RIPA buffer (50 mM HEPES-KOH, pH 7.5, 500 mM LiCl, 1 mM EDTA, 1% Igepal CA-630, 0.7% Na-deoxycholate) and once with TE (10 mM Tris-HCl, pH 8.0, 1 mM EDTA) containing 50 mM NaCl. After elution with 1% SDS-50 mM Tris-HCl pH 8.0–10 mM EDTA crosslinks were reversed by overnight incubation at 65°C. Proteinase K was added to a final concentration of 200 μg/ml for 2 h at 55°C. DNA was extracted with QIAquick PCR purification kit (QIAGEN). Obtained DNA was analyzed using real-time PCR. Significance of differences was estimated using one-way ANOVA test. The antibodies used for ChIP are presented in S3 Table. DNA obtained from ChIP reactions with antibodies to ORC4L was amplified using GenomePlex complete whole genome amplification (WGA) kit (Sigma) according to manufacturer’s instruction, negative control was also amplified.

### Real-time PCR

PCR was carried out with Taq DNA polymerase using iCycler iQ5 real-time detection system (Bio_Rad) or LightCycler 480 PCR System (Roche). Detection of amplificated products was performed using TaqMan probes. Sequences of primers and probes are given in S1 Table. The following condition were used: 95°C for 5 min and 40 cycles of 95°C for 15 s, and 56 to 60°C for 1 min. Each reaction was performed in duplicate and repeated at least for two independent DNA preparations. The amount of DNA in each independent preparation was estimated by a standard curve generated for every reaction, using a series of 10-fold dilutions of plasmid DNA for nascent DNA and 5-fold dilution of Input for DNA obtained from ChIP reactions.

### RNA isolation, reverse transcription, and PCR

RNA was isolated using TRIzol from XEN, TS cells, and fibroblasts. cDNA was synthesized using total RNA by SuperScript III (Life technologies) reverse transcriptase at 55°C according to the manufacturer’s instructions. PCR was carried out at the following parameters: 95°C for 5 min and 35 cycles of 95°C for 15 s, 58°C for 15 s, and 72°C for 30 s. Primer pairs are given in S4 Table.

### Western blotting

Western blotting was performed as described previously [28]. Total cell lysate was prepared directly in SDS-PAGE loading buffer. Western blot analysis was performed after electrophoretic separation of polypeptides by 10% SDS—PAGE and transfer to nitrocellulose membrane (Bio-Rad). Blots were probed with the primary anti-ORC4 and appropriate secondary antibodies, and detected by chemiluminescence with 1.25 mM luminol (Sigma) in 0.1 M Tris-HCl, pH 8.5, 68 mM p-Coumaric acid (Sigma) and 33% hydrogene peroxide. Antibodies were used at the following dilutions: primary anti-ORC (Abcam, ab9641) 1:500, secondary anti-rabbit IgG peroxidase conjugated (Sigma) 1:80000.

### Statistical analysis

Statistical analysis was performed using one-way ANOVA and F-test. Variances between sites were compared. The value at each site was compared with the mean value obtained from all sites in the locus using F-test. In the analysis of results obtained from ChIP with ORC4 antibodies the value at each site was compared with the values at neighboring sites using F-test.

## Supporting Information

S1 Checklist ARRIVE checklist. (PDF)

S1 Fig Western blot analysis of extracts from vole fibroblasts using ORC4 antibody—Abcam, ab9641. (TIF)

S2 Fig Distribution of histone H3 in the XIC locus. Diagram shows results of quantitative PCR analysis of ChIP with antibodies to H3 in XEN, TS cells, and fibroblasts. Legends are shown in the S2 Fig. At least two independent experiments were performed, PCR were made in duplicate. ±SD is given. Significant differences * P≥0.95 (one-way ANOVA test).
(TIF)

S3 Fig RT-PCR analysis of genes located to the vole XIC in XEN, TS cells, and fibroblasts. (TIF)

S1 Table List of primers and TaqMan probes used for real-time PCR. (DOCX)

S2 Table G4 motifs in the XIC locus of *M*. *levis*. (DOCX)

S3 Table Antibodies used for ChIP assay. (DOCX)

S4 Table List of primers used for analysis of gene expression. (DOCX)
