# Correction: Mapping of Replication Origins in the X Inactivation Center of Vole *Microtus levis* Reveals Extended Replication Initiation Zone

**DOI:** 10.1371/journal.pone.0132084

**Published:** 2015-06-26

**Authors:** Vladimir V. Sherstyuk, Alexander I. Shevchenko, Suren M. Zakian

In Fig 6, the schemes of vole and mouse X-chromosome inactivation centre are incorrectly switched, and figure legends are replaced with Russian characters. Please see the complete, correct Fig 6 and caption here.

**Fig 6:**
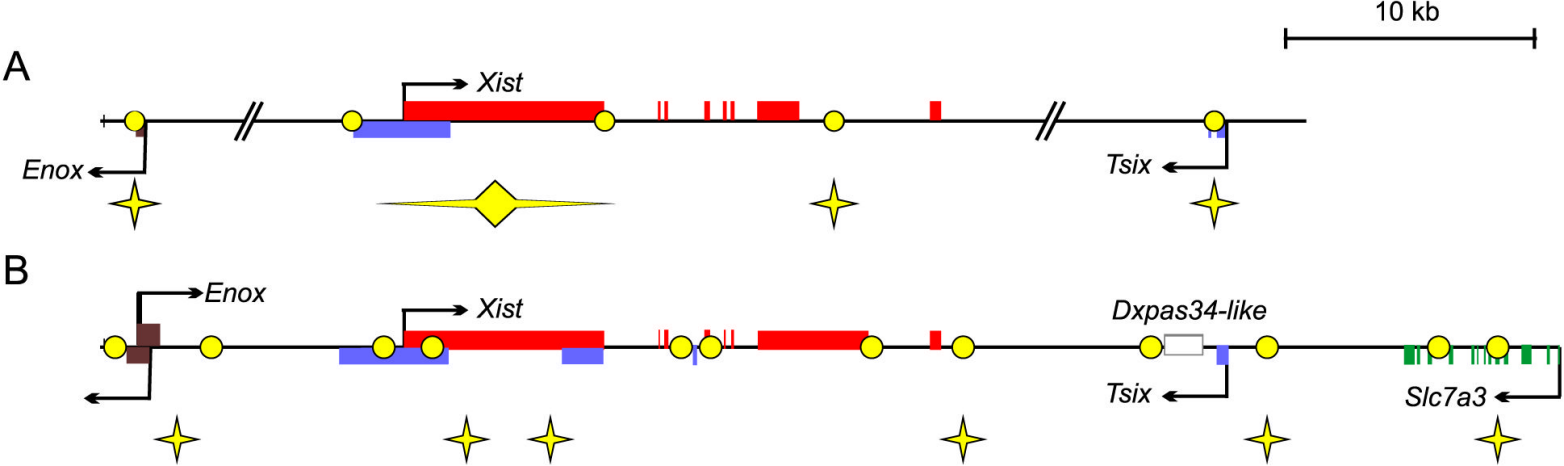
Comparative analysis of replication origin localization in the mouseand vole XICs. Schematic representation of XIC locus with identified replication origins and ORC binding regions in *M*. *musculus* (A) [30] and *M*. *levis* (B). Exons are indicated by rectangles. Arrows show direction of transcription. Stars show regions with active origins in all cell lines analyzed and circles do ORC binding regions.

## References

[1] Sherstyuk VV, Shevchenko AI, Zakian SM (2015) Mapping of Replication Origins in the X Inactivation Center of Vole *Microtus levis* Reveals Extended Replication Initiation Zone. PLoS ONE 10(6): e0128497 doi:10.1371/journal.pone.0128497 26038842 10.1371/journal.pone.0128497 PMC4454516

