# Supplementary figures and images for: Mapping of Replication Origins in the X Inactivation Center of Vole Microtus levis Reveals Extended Replication Initiation Zone
Source: PLoS One. 2015 Jun 3;10(6):e0128497. doi: 10.1371/journal.pone.0128497 (PMC4454516; doi:10.1371/journal.pone.0128497)

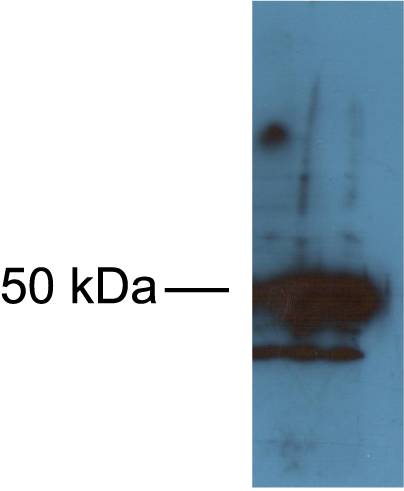

Supplement: S1 Fig — (TIF) [file pone.0128497.s002.tif]

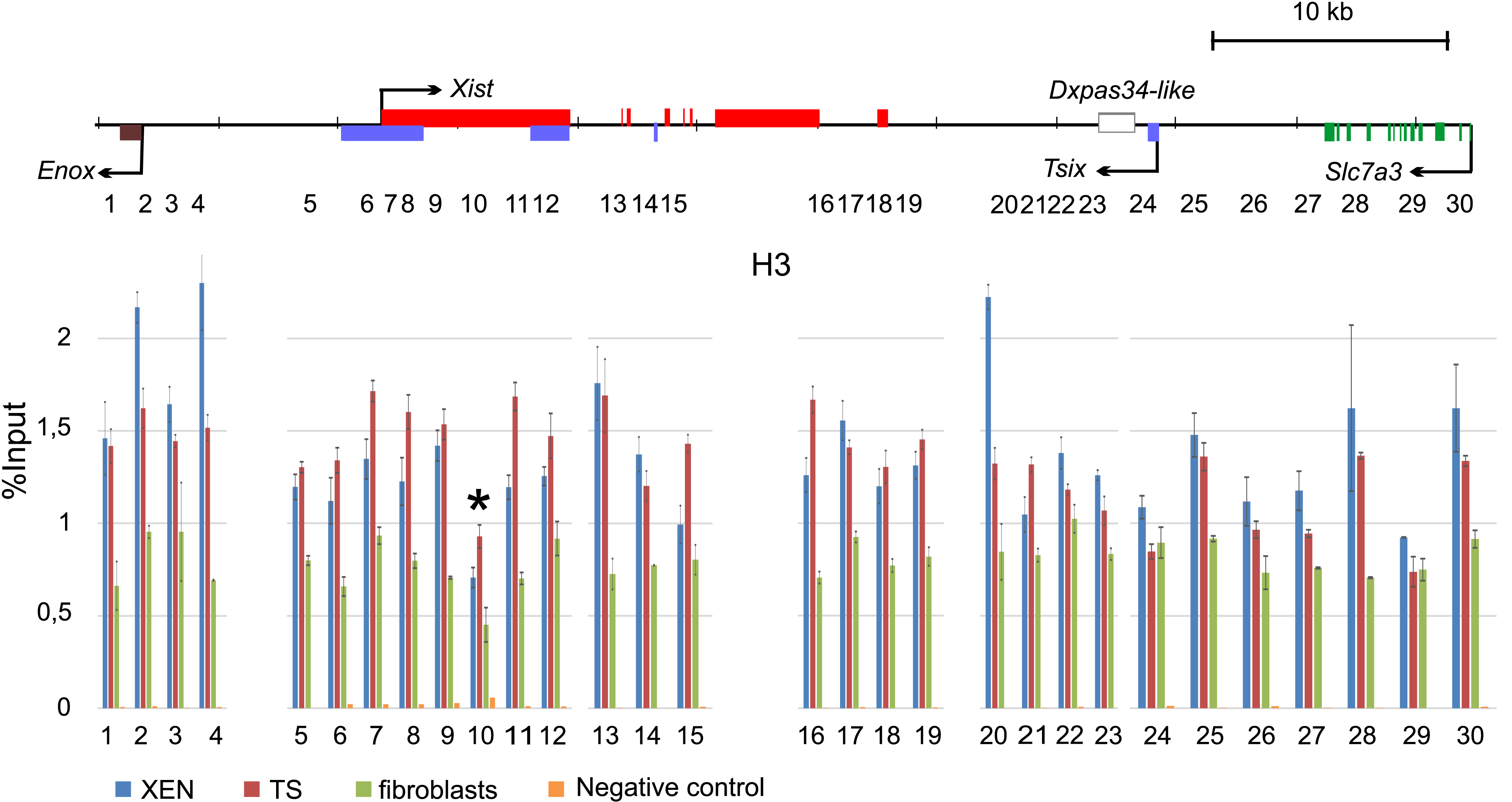

Supplement: S2 Fig — Diagram shows results of quantitative PCR analysis of ChIP with antibodies to H3 in XEN, TS cells, and fibroblasts. Legends are shown in the S2 Fig. At least two independent experiments were performed, PCR were made in duplicate. ±SD is given. Significant differences * P≥0.95 (one-way ANOVA test). (TIF) [file pone.0128497.s003.tif]

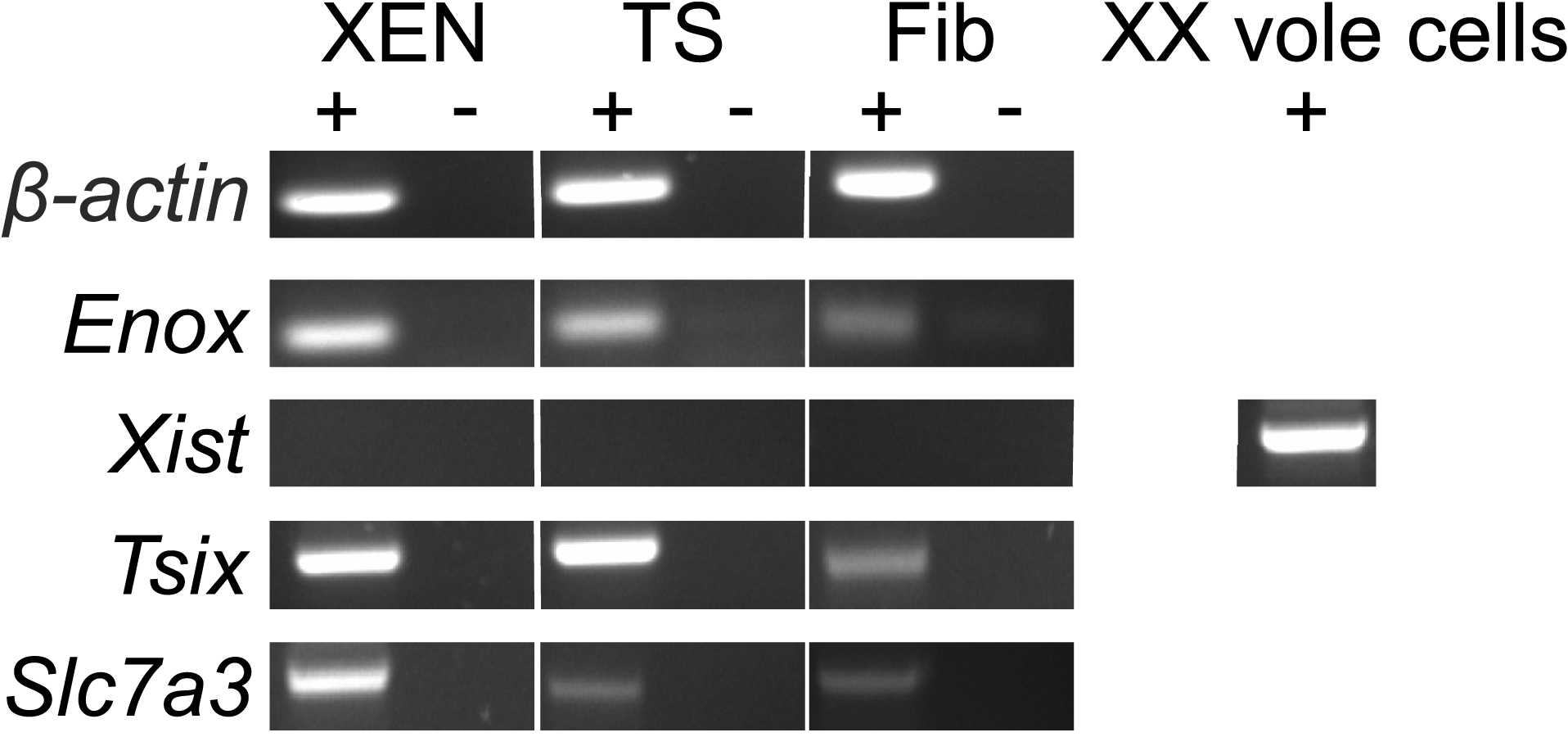

Supplement: S3 Fig — (TIF) [file pone.0128497.s004.tif]
