# Supplementary material for: Mapping of Replication Origins in the X Inactivation Center of Vole Microtus levis Reveals Extended Replication Initiation Zone
Source: PLoS One. 2015 Jun 3;10(6):e0128497. doi: 10.1371/journal.pone.0128497 (PMC4454516; doi:10.1371/journal.pone.0128497)
Supplement: S1 Table — (DOCX) [file pone.0128497.s005.docx]

**Table S1. List of primers and TaqMan probes used for real-time PCR.**

| **Site** | **Primer sequence (5’-3’)** | **TaqMan probe sequence (5’-3’)** |
| --- | --- | --- |
| 1 | ctaaaacataaagttcattttaac  agcttgttacattaagttggag | tgtcacccgatacatacatggga |
| 2 | acaaatggctacgccagcta  acacaggcagattttagagg | cggaagccgccgctgactatccg |
| 3 | acatttgtccgcttaaaggctgc  tccctcttgtgtggcaaggttac | ccctcccaatctggcagggccag |
| 4 | atcctctgggctgtgccagaagg  tgtcaggaggggagagagaa | cccactttgtctaaccggtgctcc |
| 5 | ggcatgtatgtgtaaaggtg  tggaacactgagatgtgatat | catcgccgtctgtaagatgtcca |
| 6 | cttgctagcagcggtagaga  tttcaatgaataaaattggacaatt | ctcagcggtatggcacttgccctag |
| 7 | ttccatctctgtttttgcccatg  aaaagcaggtacgtttccacagc | tgtctgtcctccttccctgcccc |
| 8 | gtgtgtgggtttggacttgat  cacataacaccacacatgaaca | tctgattccagttaccatgagtctta |
| 9 | gccgtatttagtccacactgctg  cctctggccaaggctttattctg | cccacagcaggcctggagaact |
| 10 | gagcctgggctgacacttaattc  aggacaaatgcagctgtgcacat | cgggcattgtcaccgcttctgcc |
| 11 | cccactgtgcctttcatttggac  gcctctgatgtcaaagggagtac | acgcagttgtccatccttacctttgg |
| 12 | atgaacacagcaccgatggacag  tgcttcatccacctagctttggc | tgcagcccatgtgtcccagagctcc |
| 13 | ccctataatgtagccactgc  cagatcttccccaggagtga | tcccagtccaaggagcctgttctgc |
| 14 | cctttagcgtttgacacgagga  tgctccatccttatggtccgtc | ttgcacctgcagagctccaggat |
| 15 | atgagggtcctccaggggaata  cttgccgggttcagcgaagtgt | cccggctaggactgttgcccag |
| 16 | gtcctgtccagtgtcaggta  gtattgtagtagcatctgacg | cctgtccttctgttccctgacgtcc |
| 17 | cttaggattatccctgggggaa  ggcagttctatgccttggcatgt | cccattcttgagctgcagcaggc |
| 18 | ccaatgctgacatccacaaatgg  tgtgtctgttggcccaacttagt | cccagacctcttcaacctggctc |
| 19 | gaaatggaatggactgctgctgg  ccctcctcttgtatgaggcaact | agaggccctttcctctactcagc |
| 20 | gaaataggatagaactcatcacc  gtctgaacccaccaccacac | tcttctcctgcttaacctcctgg |
| 21 | tacgtacatggaactaacactgt  ctagaggacacgtggacttctta | tccacacatgtggcaacctaggc |
| 22 | aggcatgcctgtaccagtacat  tctgtcttccactcttgtagag | catcaaaccactcaccagactgtctg |
| 23 | aacttcagggcctttcttgg  gccaaaccaaaagcatggattcg | ctcagttgcttcacatgctgccagc |
| 24 | ttcacctgtctctctgggtagcg  gcccagaacctgggacaaagtta | tttccctgctcggtttcccgaaca |
| 25 | cactttgatcagggcaggaacag  agaactccattttagacaggacc | tgctgggctgcactcacctccgc |
| 26 | atgtgaacaaggtgagaacgagc  cgtgactgggaaggttaatgagg | tcgcgactaggccctgcacactc |
| 27 | tgcagaactggcataaatactcc  gcctgtgtgtaaggaagggatag | ctccctgccctggcttcccagct |
| 28 | gcctccattcccagaagggttac  gtttggagaagcccagcttgaca | cctctctctcaccgaatgccgcc |
| 29 | ccagctttgtggctctctaacc  ccgaagaactggagcctcacaa | tgggtccacccatggtcaagca |
| 30 | acctcgctgcaggttaaaagtgg  cctaaggcaggcgggaggaagtg | ccctcctcaccaagcccacagcg |
