# Supplementary material for: Mapping of Replication Origins in the X Inactivation Center of Vole Microtus levis Reveals Extended Replication Initiation Zone
Source: PLoS One. 2015 Jun 3;10(6):e0128497. doi: 10.1371/journal.pone.0128497 (PMC4454516; doi:10.1371/journal.pone.0128497)
Supplement: S2 Table — (DOCX) [file pone.0128497.s006.docx]

**Table S2. G4 motifs in the XIC of *M. levis*.**

| **№** | **Lenght** | **G4 motif sequence** | **G-Score** |
| --- | --- | --- | --- |
| 1 | 40 | GGGAGAAGAACGGGTAGAAGGGAAACA  AAAAGGAAAAGGG | 62 |
| 2 | 27 | GGGTCTGTGGCCGGGGGAAGGGAGGGG | 65 |
| 3 | 28 | GGGGGGGGACGGGAACGTTGGATAAGGG | 62 |
| 4 | 24 | GGGGGTCGGGGACGGGGATCGGGG | 107 |
| 5 | 33 | GGGGTGGGTTGGGGTCGGGAAGCGGGG  GGGGGG | 101 |
| 6 | 19 | GGGTTGGGAACGGGACGGG | 71 |
| 7 | 21 | GGGGTCGGGGTCGGGTCGGGG | 72 |
| 8 | 30 | GGGTCCGAGATTGGGGTCCGGGAAGGA  GGG | 67 |
| 9 | 29 | GGGAAAAGGGTCGAGGTAGTGGGACAGGG | 65 |
| 10 | 33 | GGGTGAAACGTCGGGTACACAGGGTCT  CGAGGG | 69 |
| 11 | 34 | GGGTAGTGGGTGTGGGTCCGAACGGATCC  GTGGG | 60 |
| 12 | 30 | GGGAGGGTTAAAAATATGACGGGG  GGGGGG | 60 |
| 13 | 39 | GGGTGGGTGGATGGGTGGATGGATGGGTG  GATGGGTGGG | 72 |
| 14 | 15 | GGGGGGGGGGGGGGG | 72 |
| 15 | 32 | GGGATGGAAAGTGGGGAAGAAGGGGCA  GTGGG | 68 |
| 16 | 32 | GGGATTGACTAGGGTAGGAGGGAAAT  CAAGGG | 69 |
| 17 | 27 | GGGTGGGGGATGGGGTCTGGATGAGGG | 66 |
| 18 | 24 | GGGACCAGGGACCTGGGACCAGGG | 72 |
| 19 | 26 | GGGGTGGGTAAAGACGGGGACTGGGG | 67 |
| 20 | 28 | GGGCTCGGGGAAGTAAGGGGATGCTGGG | 70 |
| 21 | 26 | GGGGAGAGGGAGAGAGGGAGAGAGGG | 71 |
| 22 | 38 | GGGTAAGACCTAAGGGAGAGGAGGGGGT  ACTCCCAGGG | 70 |
| 23 | 35 | GGGAAGGTCCGGGTCGGGGCGGGTAGT  GAGTTGGG | 70 |
| 24 | 28 | GGGATGAGGTGGGGGGGGTAGCGTGGGG | 67 |
| 25 | 15 | GGGCGGGAGGGGGGG | 72 |
