# Supplementary material for: Mapping of Replication Origins in the X Inactivation Center of Vole Microtus levis Reveals Extended Replication Initiation Zone
Source: PLoS One. 2015 Jun 3;10(6):e0128497. doi: 10.1371/journal.pone.0128497 (PMC4454516; doi:10.1371/journal.pone.0128497)
Supplement: S3 Table — (DOCX) [file pone.0128497.s007.docx]

**Table S3. Antibodies used in ChIP assay.**

| **Antibodies** | **Raised/Type** | **Source** | **Cat. No.** | **Dilution** |
| --- | --- | --- | --- | --- |
| Anti ORC4L | Goat polyclonal | Abcam | ab9641 | 1:250 |
| Anti H3 | Rabbit monoclonal | Upstate (Millipore) | 05-928 | 1:250 |
| Anti H3.3 | Rabbit polyclonal | Upstate (Millipore) | 09-838 | 1:250 |
| Anti H3K9ac | Rabbit polyclonal | Abcam | ab4441 | 1:125 |
| Anti H4K20me1 | Rabbit polyclonal | Abcam | ab9051 | 1:100 |
| Anti H3K27me3 | Rabbit monoclonal | Upstate (Millipore) | 07-449 | 1:250 |
