# Supplementary material for: Mapping of Replication Origins in the X Inactivation Center of Vole Microtus levis Reveals Extended Replication Initiation Zone
Source: PLoS One. 2015 Jun 3;10(6):e0128497. doi: 10.1371/journal.pone.0128497 (PMC4454516; doi:10.1371/journal.pone.0128497)
Supplement: S4 Table — (DOCX) [file pone.0128497.s008.docx]

| Gene | Sequence (5’-3’) | T_a_ |
| --- | --- | --- |
| *β-actin* | F GATATCGCTGCGCTGGTCGT  R AGATCTTCTCCATGTCGTCC | 60˚C |
| *Enox* | F ACAAATGGCTACGCCAGCTA  R ACACAGGCAGATTTTAGAGG | 60˚C |
| *Xist* | F TTGCTCAGATTAGCTAG  R GTGATTAATTCATTCTATCTGC | 48˚C |
| *Tsix* | F CCATGTGACATTGCTGATGAAACC  R CTCTCCCTGCGCTCCCTCAC | 60˚C |
| *Slc7a3* | F GTCTGGGTAGGGTTTGATGATTCT  R CCATTGTGGCCACTGTGGTATCT | 60˚C |
